# Emergence of carbapenem-resistant *Salmonella* Typhi harboring *bla*_NDM-5_ in India: genomic evidence from a multicenter study

**DOI:** 10.3389/fmicb.2025.1685068

**Published:** 2025-12-03

**Authors:** Tharani Priya Thirumoorthy, Jobin John Jacob, Monisha Priya Teekaraman, S. Mahantesh, Bhavana Jagannatha, Suhani Manasa, Savitha Nagaraj, Jayanthi Savio, Priyadarshini A. Padaki, J. Sudarsana, Ashalatha Nair, Sukanya Verma, Raman Gaikwad, Divya Joshi, Vasant C. Nagvekar, Camilla Rodrigues, Pavithra Sathya Narayanan, Aravind Velmurugan, K. B. Santhosh, Jacob John, Kamini Walia, Balaji Veeraraghavan

**Affiliations:** 1 Christian Medical College, Vellore, Tamil Nadu, India; 2 The Tamil Nadu Dr. M.G.R. Medical University, Chennai, Tamil Nadu, India; 3 Indira Gandhi Institute of Child Health, Bengaluru, Karnataka, India; 4 St. John’s Medical College Hospital, Bengaluru, Karnataka, India; 5 Baby Memorial Hospital, Kozhikode, Kerala, India; 6 KIMSHEALTH Hospital, Thiruvananthapuram, Kerala, India; 7 Agilus Diagnostic Labs, Mumbai, Maharashtra, India; 8 Sahyadri Speciality Labs, Pune, Maharashtra, India; 9 Fortis Hospital, Bengaluru, Karnataka, India; 10 Lilavati Hospital & Research Centre, Mumbai, India; 11 P. D. Hinduja Hospital & Medical Research Centre, Mumbai, India; 12 Descriptive Research Division, Indian Council of Medical Research, New Delhi, India

**Keywords:** *Salmonella* Typhi, enteric fever, carbapenem-resistant, whole genome sequencing, outbreak investigation

## Abstract

**Background:**

The rise of antimicrobial resistance (AMR) in *Salmonella enterica* serovar Typhi poses a serious threat to global enteric fever control. In particular, the emergence of resistance to third-generation cephalosporins and azithromycin critically undermines available treatment options. Sustained genomic surveillance of high-risk *S*. Typhi lineages and resistance determinants is essential for informing antibiotic policy and optimizing typhoid conjugate vaccine (TCV) introduction in endemic regions. In this study, we report a multicenter outbreak of carbapenem-resistant *S*. Typhi in India and investigate its genomic epidemiology, resistance mechanisms, and evolutionary origins.

**Methods:**

A total of 31 carbapenem-resistant *S*. Typhi isolates collected from multiple tertiary care hospitals were subjected to phenotypic antimicrobial susceptibility testing and whole-genome sequencing (WGS). Short-read WGS data were used to analyze core-genome SNPs, infer phylogenetic relationships, and investigate AMR determinants. Two representative isolates underwent long-read Oxford Nanopore sequencing for plasmid reconstruction and comparative genomic analysis with Enterobacterales.

**Results:**

Antimicrobial susceptibility testing of isolates revealed resistance to ampicillin, ciprofloxacin, ceftriaxone, and carbapenems while retaining susceptibility to chloramphenicol, cotrimoxazole, and azithromycin. The genomic analysis identified the presence of two plasmids: IncFIB(K) harboring *bla*_CTX-M-15_, *qnrS1*, *tetA*, and IncX3, carrying the *bla*_NDM-5_ gene. Phylogenetic analysis classified the isolates within a novel genotype, 4.3.1.1.1, belonging to genotype 4.3.1.1 (H58 lineage I). Notably, plasmid comparison revealed high similarity to resistance plasmids circulating in co-endemic *Escherichia coli* and *Klebsiella pneumoniae*, indicating recent horizontal gene transfer.

**Conclusion:**

This is the first documented outbreak of *bla*_NDM_-mediated carbapenem-resistant *S*. Typhi, highlighting a new stage in the evolution of drug-resistant typhoid. The acquisition of high-risk plasmids by *S*. Typhi and their integration into successful epidemic lineages underscores the urgent need for strengthened genomic surveillance and inter-species AMR tracking. Our findings have direct implications for treatment guidelines, TCV implementation strategies, and efforts to prevent global dissemination of carbapenem-resistant *S*. Typhi.

## Introduction

Enteric fever, primarily caused by *Salmonella enterica* serovars Typhi and Paratyphi A, remains a major public health challenge, particularly in low- and middle-income countries (LMICs) (Parry et al., 2002; Crump et al., 2015). This disease is primarily transmitted through the ingestion of food or water contaminated with fecal matter, potentially leading to severe complications, such as intestinal perforation, if untreated (Radhakrishnan et al., 2018; Crump, 2019). Recent estimates indicate a global burden of approximately 9.24 million enteric fever cases in 2021,^1^ with South Asia accounting for 62% of the total incidence. Within this region, India bears the highest burden, contributing 58% of global cases, followed by Pakistan and Bangladesh (Piovani et al., 2024). Notably, children under 5 years of age are disproportionately affected, experiencing a significant share of the disease’s morbidity and mortality, particularly in endemic areas (John et al., 2023).

The management of typhoid fever has historically relied on antimicrobial therapy; however, the emergence of antimicrobial-resistant (AMR) strains has significantly compromised treatment efficacy (Browne et al., 2024). In the late 20th century, multidrug-resistant (MDR) *S*. Typhi strains emerged, exhibiting resistance to first-line antibiotics such as ampicillin, chloramphenicol, and trimethoprim-sulfamethoxazole (Crump et al., 2015). This situation prompted a shift towards fluoroquinolones as the primary treatment option (Marchello et al., 2020). Unfortunately, the subsequent rise of fluoroquinolone non-susceptible (FQNS) strains necessitated the exploration of alternative therapies, including azithromycin and third-generation cephalosporins (Kirchhelle et al., 2019). In recent years, the emergence and spread of ceftriaxone-resistant *S*. Typhi strains in Pakistan and India has further complicated the treatment strategies. In Pakistan, the outbreak of extensively drug-resistant (XDR) *S*. Typhi, has spread across the country, with ~5,274 cases reported by December 2018 (Akram et al., 2020). These XDR strains exhibit resistance to first-line antibiotics, fluoroquinolones, and third-generation cephalosporins, leaving azithromycin as the primary treatment options (Akram et al., 2020; Klemm et al., 2018). Similarly, ceftriaxone-resistant *S*. Typhi outbreaks have been documented in India, including in Mumbai and Vadodara (Jacob et al., 2021; Thirumoorthy et al., 2025). Furthermore, azithromycin-resistant strains, driven by mutations in the *acrB* gene, threaten one of the few remaining oral treatment options, highlighting the urgent need for new therapeutic strategies (Carey et al., 2021).

Genomic surveillance is a key tool for monitoring AMR transmission and tracking outbreaks of *S*. Typhi (Baker et al., 2018). The GenoTyphi scheme provides a robust framework for classifying *S*. Typhi into four major lineages and over 75 genotypes, enabling the identification of distinct transmission pathways on a global scale (Wong et al., 2016; Dyson and Holt, 2021). Among these, haplotype 58 (H58, genotype 4.3.1) has become the dominant strain worldwide, primarily due to its enhanced transmissibility and MDR (Wong et al., 2015; Pragasam et al., 2020; Carey et al., 2024). Within this lineage, key subclades include 4.3.1.1, associated with MDR, 4.3.1.2, linked to FQNS, and 4.3.1.3, which predominates in Bangladesh, providing critical epidemiological insights into the evolution and spread of drug-resistant typhoid (Carey et al., 2023). Further the GenoTyphi framework has proven effective in managing outbreaks of drug-resistant *S*. Typhi. For instance, the XDR *S*. Typhi outbreak in Pakistan, attributed to subclade 4.3.1.1.P1, was tracked using this scheme (Klemm et al., 2018). Similarly, ceftriaxone-resistant *S*. Typhi outbreaks in India, including in Mumbai (subclade 4.3.1.2.1) and Vadodara (4.3.1.2.2) were effectively tracked and characterized (Jacob et al., 2021; Thirumoorthy et al., 2025; Argimón et al., 2022).

Recently, sporadic reports have highlighted the emergence of carbapenem-resistant *S*. Typhi (CRST) in Pakistan, raising serious concerns about the evolving landscape of antimicrobial resistance (AMR) in this pathogen (Ain et al., 2022; Nizamuddin et al., 2023). More alarmingly, similar cases have been reported in various cities across southern and western India, indicating a potential regional spread of this resistance profile in a region where typhoid fever remains endemic. Despite these developments, the genomic epidemiology and evolutionary trajectory of CRST strains in India remain poorly understood. In this study, we aimed to investigate the genomic characteristics and evolutionary dynamics of CRST isolates in India, with particular focus on their rising prevalence and the role of plasmids in mediating resistance (Tharani Priya et al., 2025). A deeper understanding of these factors is critical to inform public health strategies and guide targeted interventions to curb the spread of CRST in endemic settings.

## Methods

### Study settings

A total of 32 *Salmonella* Typhi isolates were obtained between August 2024 and May 2025 from enteric fever patients treated at hospitals in various cities in South and West India and from individuals with recent travel exposure to these regions. Among the isolates, sixteen originated from St. John’s Medical College Hospital, Bengaluru, five from Fortis Hospital, Bengaluru, four from Indira Gandhi Institute of Child Health, Bengaluru, two from Baby Memorial Hospital, Kozhikode and one each from Agilus Diagnostics Lab, Mumbai, KIMS Health Hospital, Trivandrum, P. D. Hinduja Hospital and Medical Research Centre, Mumbai, Christian Medical College, Chittoor campus, Andhra Pradesh and Sahyadri Speciality Labs, Pune, Maharashtra. Participating institutions flagged these isolates due to atypical antimicrobial resistance profiles detected via phenotypic susceptibility testing and/or automated VITEK 2 systems. Detailed patient information, including clinical affiliations and travel histories, is provided in Supplementary Table S1. For in-depth analysis of resistance mechanisms, all isolates were transferred to the Department of Clinical Microbiology at Christian Medical College (CMC), Vellore, for whole-genome sequencing and genomic characterization.

### Bacterial isolates and phenotypic testing

The isolates, once received at CMC Vellore, was cultured on blood and MacConkey agar plates to ensure purity. The isolates were confirmed *S*. Typhi by conventional biochemical tests, serotyping (Kauffmann–White scheme), and qPCR (Nair et al., 2019). Antimicrobial susceptibility (AST) of the study isolates were evaluated by Kirby–Bauer disk diffusion technique, with inhibition zone measurements and interpretations adhering to the Clinical and Laboratory Standards Institute criteria (Clinical and Laboratory Standards Institute, 2024). The tested antibiotics included ampicillin (10 μg), chloramphenicol (30 μg), trimethoprim/sulfamethoxazole (1.25/23.75 μg), ciprofloxacin (5 μg), pefloxacin (5 μg), ceftriaxone (30 μg), cefixime (5 μg), azithromycin (15 μg), meropenem (10 μg), and ertapenem (10 μg). To complement these findings, the broth microdilution (BMD) method was employed to assess the minimum inhibitory concentrations (MICs) of additional antibiotics, namely cefepime, aztreonam, piperacillin-tazobactam (fixed 4 μg/mL), ceftazidime-avibactam (fixed 4 μg/mL), aztreonam-avibactam (fixed 4 μg/mL), and colistin. Colistin MIC was interpreted according to EUCAST guidelines (The European Committee on Antimicrobial Susceptibility Testing, 2024).

### DNA extraction and whole genome sequencing (WGS)

Genomic DNA was isolated from samples using the QIAamp^®^ Mini Kit (250) (QIAGEN, Hilden, Germany) according to the manufacturer’s protocol. DNA purity and concentration were quantified using a Nanodrop One spectrophotometer (Thermo Fisher Scientific, Waltham, United States) and a Qubit Fluorometer with the dsDNA HS Assay Kit (Life Technologies, Carlsbad, United States). The presence of carbapenemase and ESBL genes were identified by multiplex PCR with an in-house gene panel, adapted from the methodology described by Poirel et al. (2011).

For short-read sequencing, DNA was fragmented, and paired-end libraries were prepared using the Illumina Nextera DNA Flex Library Kit and Nextera DNA CD Indexes (Illumina, Massachusetts, United States). Equimolar library pools were sequenced on the Illumina NovaSeq 6000 platform (available at Unipath Specialty Laboratory Limited, Ahmedabad, India), generating 2 × 150 bp paired-end reads. All steps, including tagmentation, library amplification, and purification, were performed as specified by the manufacturer.

To complement the short-read data, long-read sequencing was performed on two isolates using Oxford Nanopore Technology (ONT). For each sample, approximately 200 ng of DNA was processed with the Nanopore Rapid Barcoding Kit 96 V14 (SQK-RBK114.96; Oxford Nanopore Technologies, Oxford, United Kingdom) following the manufacturer’s protocol. Sequencing was carried out on a PromethION P2 Solo platform with real-time base-calling enabled during the run. Basecalling of POD5 files was executed using Dorado v0.8.3 (Oxford Nanopore Technologies) with the dna_r10.4.1_e8.2_400bps_sup@v5.0.0 model to ensure high-accuracy sequence reconstruction.

### Quality control, assembly and annotation

Quality assessment of Illumina reads was performed using FastQC v0.12.1^2^ followed by adapter and index trimming with Trimmomatic v0.39 (Bolger et al., 2014). Contaminant screening and filtering were executed with Kraken v1.1.1 (Wood et al., 2019),^3^ followed by sequence coverage analysis. High-quality reads (Phred score >30) were assembled into draft genomes using SKESA (Souvorov et al., 2018).^4^ For hybrid assembly, the Hybracter pipeline v0.8.0 (Bouras et al., 2024)^5^ was employed to process Oxford Nanopore (ONT) long reads. This workflow first improved the read quality with Filtlong, assembled long reads *de novo* using Flye, and polished the assemblies iteratively with Medaka. Further refinement was achieved by polishing Illumina short reads via Polypolish and PyPolca. Final genome completeness and accuracy were evaluated using QUAST v5.2.0 (Gurevich et al., 2013). Genome annotations were performed using Bakta v1.10.1 (Schwengers et al., 2021).^6^ Unless specified, default parameters were applied throughout all analytical steps.

### Comparative genome analysis

Draft genome assemblies were analyzed with SeqSero v2.0 (Zhang et al., 2019)^7^ to verify the antigenic composition of the serotype. *In silico* multilocus sequence typing (MLST) (Larsen et al., 2012) was performed on all isolates using the pipeline provided by the Center for Genomic Epidemiology.^8^ Antimicrobial resistance (AMR) genes were identified using NCBI AMRFinderPlus v4.0.3 (Feldgarden et al., 2021).^9^ Plasmid content was determined by querying genome sequences against the PlasmidFinder database (Carattoli et al., 2014).^10^ Plasmids were compared using the Basic Local Alignment Search Tool (BLAST), and circular maps were prepared using the Proksee server^11^ (Grant et al., 2023).

### Genotyping and phylogeny

The isolates were assigned to previously defined genotypes using the GenoTyphi pipeline (available at: https://github.com/katholt/genotyphi). Unique single nucleotide polymorphisms (SNPs) characterizing the novel sub-lineage were and subsequently incorporated into the GenoTyphi framework to track the outbreak in future investigations.

For phylogenetic analysis, genome assemblies of *S*. Typhi (*n* = 412) representing all major genotypes were obtained from the curated global collection provided by the Global Typhoid Genomics Consortium^12^ (Carey et al., 2023), with data sourced from NCBI (Supplementary Table S2). The sequencing reads were aligned to the reference genome of *S*. Typhi CT18 (GenBank: AL513382.1) using Snippy v4.6.0.^13^ The resulting full alignment was first filtered to exclude SNPs located within the 354 kb of repetitive regions in the *S*. Typhi CT18 reference chromosome, using previously established coordinates (Wong et al., 2015) processed using Gubbins v3.3.3 (Croucher et al., 2015). After masking these regions, the alignment was processed with Gubbins^14^ to remove recombination sites (Wood et al., 2019). SNPs were extracted using SNP-sites v2.5.1^15^ (Page et al., 2016). A maximum-likelihood phylogeny was then reconstructed from the filtered alignment using the TVM + F + ASC + G4 model with 1,000 bootstraps using IQ-TREE v.2.4.0 (Nguyen et al., 2015) integrated with ModelFinder (Kalyaanamoorthy et al., 2017). The resulting phylogenetic tree was visualized and annotated using the Interactive Tree of Life software (iTOL v.5) (Letunic and Bork, 2021).

## Results

### Identification of CRST isolates and resistance profile

A total of 32 non-duplicated *S*. Typhi isolates were included in this study, collected from patients diagnosed with typhoid fever across multiple healthcare institutions in southern and western India, between 2024 and 2025. All isolates were confirmed as *S*. Typhi through standard biochemical tests, conventional serotyping methods, and qPCR. AST by disk diffusion revealed a consistent resistance profile across all *Salmonella* Typhi isolates except one, with resistance to ampicillin, ciprofloxacin, ceftriaxone, and carbapenems. One isolate (ERR15187853) was resistant to ampicillin, ciprofloxacin, and ceftriaxone but susceptible to carbapenems. Conversely, all isolates remained susceptible to chloramphenicol, trimethoprim-sulfamethoxazole, and azithromycin.

MIC testing confirmed high-level resistance to ciprofloxacin, ceftriaxone, ertapenem, and meropenem, with retained susceptibility to chloramphenicol, trimethoprim-sulfamethoxazole, azithromycin, and aztreonam-avibactam. MIC values for all antibiotics tested, including β-lactam/β-lactamase inhibitor combinations and colistin, are provided in Table 1. The multiplex PCR analysis confirmed the presence of both *bla*_NDM_ and *bla*_CTX-M_ genes in 31 CRST isolates, while the remaining isolate carried *bla*_CTX-M_ alone. This indicates the predominance of co-occurring carbapenemase (*bla*_NDM_) and extended-spectrum β-lactamase (*bla*_CTX-M_) genes among CRST strains.

**Table 1:** Antimicrobial susceptibility profile and minimum inhibitory concentration (MIC) in μg/mL of different antibiotics against *S*. Typhi isolates.

Isolates ID	CHL^b^	SXT^b^	CIP^b^	CTR^b^	AZI^b^	FEP^a^	ATM^a^	TZP^a^	CZA^a^	ATM-AVI^c^	COL^a^
ERR14867532 (B2288)	4 S	≤0.12 S	2 R	>1,024 R	4 S	>128 R	>128 R	>128 R	64 R	0.06 S	0.12 I
ERR14867535 (BT-10862)	4 S	0.12 S	2 R	>1,024 R	4 S	>128 R	>128 R	>128 R	64 R	0.03 S	0.015 I
ERR14867539 (BT-12236)	2 S	0.12 S	4 R	>1,024 R	2 S	128 R	128 R	128 R	64 R	0.06 S	0.12 I
ERR14867533 (B5777)	4 S	≤0.12 S	2 R	>1,024 R	4 S	>128 R	>128 R	>128 R	32 R	0.12 S	0.015 I
ERR14867544 (B5971)	4 S	≤0.12 S	1 R	>1,024 R	2 S	>128 R	>128 R	>128 R	32 R	0.06 S	0.008 I
ERR14867538 (BT14177)	1 S	≤0.12 S	1 R	>1,024 R	2 S	>128 R	>128 R	>128 R	32 R	0.06 S	0.015 I
ERR14867545 (BC248)	2 S	≤0.12 S	1 R	>1,024 R	2 S	>128 R	>128 R	>128 R	32 R	0.06 S	0.015 I
ERR14867543 (PDH2)	4 S	≤0.12 S	1 R	>1,024 R	2 S	>128 R	>128 R	>128 R	32 R	0.06 S	0.015 I
ERR14867534 (BT-15499)	4 S	0.12 S	1 R	>1,024 R	2 S	>128 R	>128 R	>128 R	32 R	0.03 S	0.015 I
ERR14867537 (BT15401)	4 S	≤0.12 S	1 R	>1,024 R	2 S	>128 R	>128 R	>128 R	32 R	0.03 S	0.015 I
ERR14867546 (BT-15515)	2 S	≤0.12 S	1 R	>1,024 R	2 S	>128 R	>128 R	>128 R	32 R	0.03 S	0.015 I
ERR14867536 (BT-15709)	4 S	≤0.12 S	1 R	>1,024 R	2 S	>128 R	>128 R	>128 R	32 R	0.06 S	0.008 I
ERR14867541 (BT-15985)	2 S	≤0.12 S	1 R	>1,024 R	2 S	>128 R	>128 R	>128 R	64 R	0.06 S	0.008 I
ERR15187847 (ADLM-01)	4 S	≤0.12 S	2 R	>1,024 R	2 S	>128 R	>128 R	>128 R	32 R	0.03 S	0.015 I
ERR14867540 (BT-17157)	4 S	≤0.12 S	2 R	>1,024 R	2 S	>128 R	>128 R	>128 R	32 R	0.03 S	0.015 I
ERR14867542 (FB01)	2 S	0.12 S	1 R	>1,024 R	2 S	>128 R	>128 R	>128 R	32 R	0.06 S	0.015 I
ERR15187850 (BT-1949)	4 S	≤0.12 S	2 R	>1,024 R	2 S	>128 R	>128 R	>128 R	32 R	0.06 S	0.015 I
ERR15187848 (BA4920)	4 S	≤0.12 S	1 R	>1,024 R	2 S	>128 R	>128 R	>128 R	64 R	0.06 S	0.008 I
ERR15187849 (480/16319)	4 S	≤0.12 S	1 R	>1,024 R	2 S	>128 R	>128 R	>128 R	32 R	0.03 S	0.015 I
ERR15187851 (336/11763)	2 S	0.12 S	1 R	>1,024 R	2 S	>128 R	>128 R	>128 R	32 R	0.03 S	0.015 I
ERR15187852 (FB02)	4 S	0.12 S	1 R	>1,024 R	2 S	>128 R	>128 R	>128 R	32 R	0.03 S	0.015 I
ERR15187853 (BT-17019)	2 S	≤0.12 S	2 R	>1,024 R	2 S	128 R	>64 R	4 S	0.5 S	2 S	0.008 I
ERR15663529 (BT0149A)	2 S	≤0.12 S	2 R	>1,024 R	4 S	>128 R	>128 R	>128 R	32 R	0.12 S	0.015 I
ERR15663527 (BC-29)	2 S	≤0.12 S	1 R	>1,024 R	2 S	>128 R	>128 R	>128 R	32 R	0.06 S	0.015 I
ERR15663528 (BC-474)	4 S	≤0.12 S	1 R	>1,024 R	2 S	>128 R	>128 R	>128 R	32 R	0.06 S	0.015 I
ERR15663530 (BT-4233)	2 S	≤0.12 S	2 R	>1,024 R	2 S	>128 R	>128 R	>128 R	32 R	0.03 S	0.015 I
ERR15663532 (BT-4326)	4 S	≤0.12 S	2 R	>1,024 R	2 S	>128 R	>128 R	>128 R	32 R	0.03 S	0.015 I
ERR15663531 (BT-4277)	4 S	≤0.12 S	2 R	>1,024 R	4 S	>128 R	>128 R	>128 R	32 R	0.12 S	0.015 I
ERR15663526 (B-1295)	2 S	0.12 S	1 R	>1,024 R	2 S	>128 R	>128 R	>128 R	32 R	0.06 S	0.015 I
ERR15663535 (FB03)	4 S	≤0.12 S	2 R	>1,024 R	4 S	>128 R	>128 R	>128 R	64 R	0.06 S	0.12 I
ERR15663533 (BT-5665)	2 S	0.12 S	1 R	>1,024 R	2 S	>128 R	>128 R	>128 R	32 R	0.06 S	0.015 I
ERR15663534 (BT-6355)	2 S	0.12 S	1 R	>1,024 R	2 S	>128 R	>128 R	>128 R	32 R	0.06 S	0.015 I

CIP, ciprofloxacin; CTR, ceftriaxone; AZI, azithromycin; FEP, cefepime; ATM, aztreonam; TZP, piperacillin-tazobactam; CZA, ceftazidime-avibactam; ATM-AVI, aztreonam-avibactam; COL, colistin.

a Interpretation based on CLSI 2024 guidelines (Enterobacteriales excluding Salmonella and Shigella).

b Interpretation based on CLSI 2024 guidelines (Enterobacteriales including Salmonella and Shigella).

c Interpretation based on EUCAST v.14.

### Genotyping and comparative genome analysis

The *S*. Typhi genomes (*n* = 32) were characterized using the GenoTyphi genotyping scheme (Wong et al., 2016; Dyson and Holt, 2021). All study isolates were identified as belonging to the H58 haplotype (genotype 4.3.1), specifically falling within the 4.3.1.1 genotype (H58 Lineage I) (Supplementary Figure S1). Based on their shared genomic features and epidemiological significance related to carbapenem resistance, these isolates have been assigned to a novel sub-genotype within 4.3.1.1, designated as 4.3.1.1.1.

AMR gene profiling identified the presence of *bla*_NDM-5_, *bla*_CTX-M-15_, *qnrS*, and *tetA* among the isolates. In addition, resistance-associated point mutation analysis revealed an S83Y substitution in *gyrA* within the quinolone resistance-determining region (QRDR). The presence of *bla*_NDM-5_ correlates with resistance to carbapenems, *bla*_CTX-M-15_ confers resistance to third-generation cephalosporins (3GCs), *tetA* is linked to tetracycline resistance, while both the S83Y mutation in *gyrA* and *qnrS* contribute to fluoroquinolone resistance. Among the two plasmids identified IncFIB(K) carried *bla*_CTX-M-15_, *qnrS*, and *tetA*, while *bla*_NDM-5_ was harbored by IncX3 plasmid.

### Population structure of CRST isolates from India

**Figure 1:**
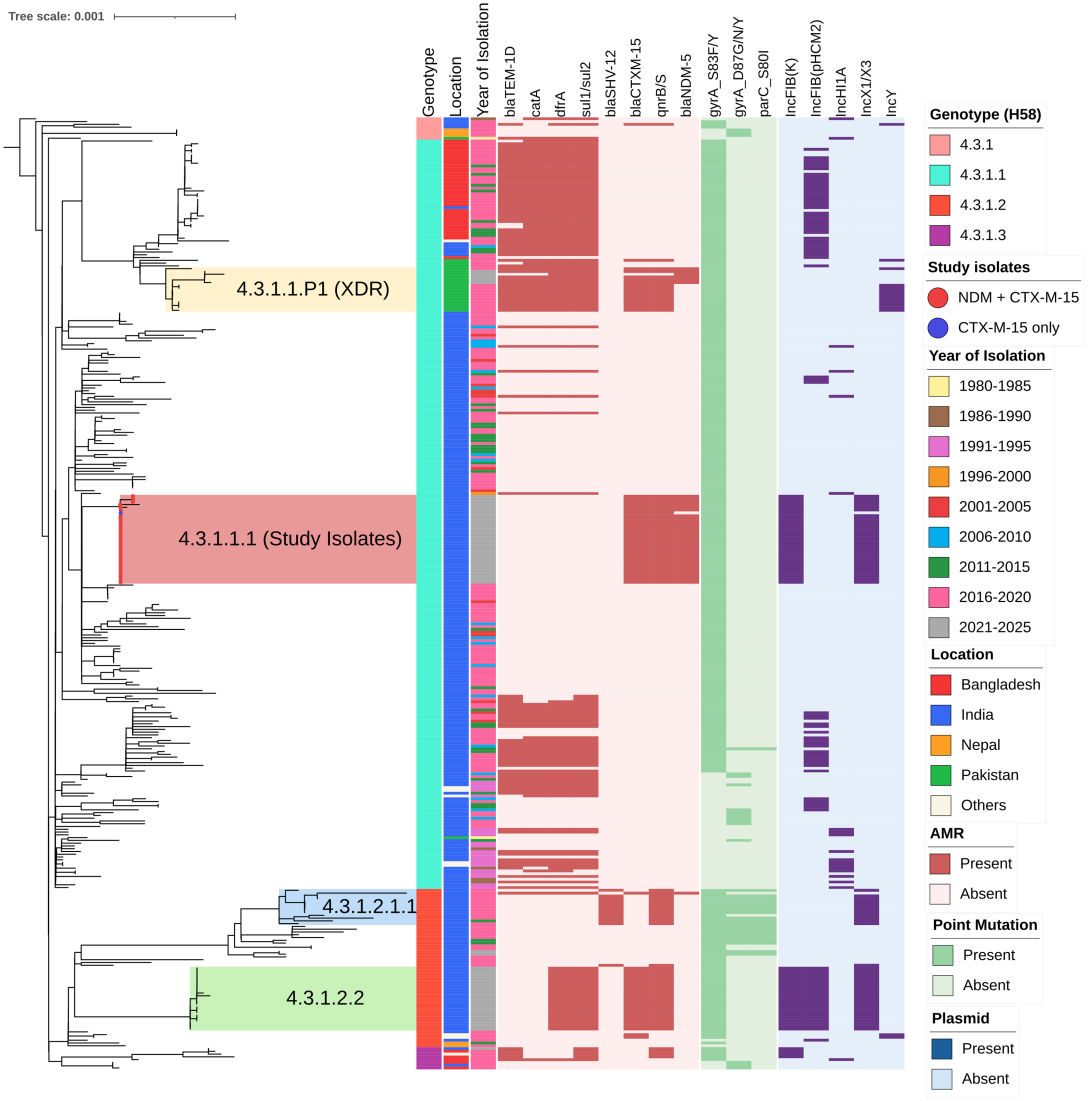
Phylogenetic relationship and genetic characteristics of 32 *S*. Typhi strains from India. A maximum likelihood phylogenetic tree was constructed using core genome SNPs from the study isolates (marked with red circles at the branch tips) along with 311 global H58 isolates, revealing an overall SNP difference of 1,149 among the H58 isolates. The tree is rooted against outgroup isolate belonging to genotype 4.3.1 (ERR1764570) and inferred using IQ-TREE2 (http://www.iqtree.org/), with bootstrap support values calculated from 1,000 replicates. Previously reported high-risk clones of cephalosporin-resistant clusters in the tree, including 4.3.1.2.1.1 (blue), 4.3.1.1.P1 (yellow), and 4.3.1.2.2 (green). The color-coded metadata strips adjacent to the tree represent: strip 1 denotes genotype, strip 2 indicates the country location, and strip 3 shows the year of isolation of each isolate. The heatmap alongside the tree depicts the distribution of antimicrobial resistance (AMR) genes, point mutations, and plasmid replicons, which are predominantly concentrated within the H58 cluster. The scale bar indicates substitutions per site. The tree was visualized and annotated using iTOL (https://itol.embl.de/).

A core genome SNP-based phylogenetic analysis, incorporating the 31 CRST study isolates and 311 global reference isolates, revealed that the CRST isolates formed a distinct subclade within the H58 lineage I (genotype 4.3.1.1) (Figure 1). This CRST subclade was distinguished from its parent clade by seven unique SNPs, underscoring its genetic distinctiveness (Supplementary Table S3). Notably, the CRST isolates exhibited the closest genetic relatedness (10 SNP difference) to a previously sequenced cluster of six *S*. Typhi isolates from India, identified through the Surveillance for Enteric Fever in India (SEFI) study. These six isolates were collected from two geographic locations Anantapur, Andhra Pradesh (ERR4790795, ERR5200930, ERR4790761, ERR5201325, ERR5201273) and Bengaluru, Karnataka (ERR5200874). All isolates were sequenced between 2018 and 2020 as part of the SEFI initiative (Supplementary Figure S2). Isolates closely related to the CRST clone but lacking resistance plasmids have been circulating in India since at least 2018, indicating a pre-existing susceptible lineage that subsequently acquired plasmid-mediated resistance determinants. This finding suggests that the CRST clone likely evolved locally from these endemic lineages through the recent acquisition of resistance plasmids. Furthermore, the CRST isolates sequenced in this study were phylogenetically distinct from both the previously reported CRST isolate from Pakistan (SRR22801806; 35 SNPs difference) and the ceftriaxone-resistant outbreak isolates recently identified in Gujarat, India (31 SNPs difference).

### Characterization of plasmids

**Figure 2:**
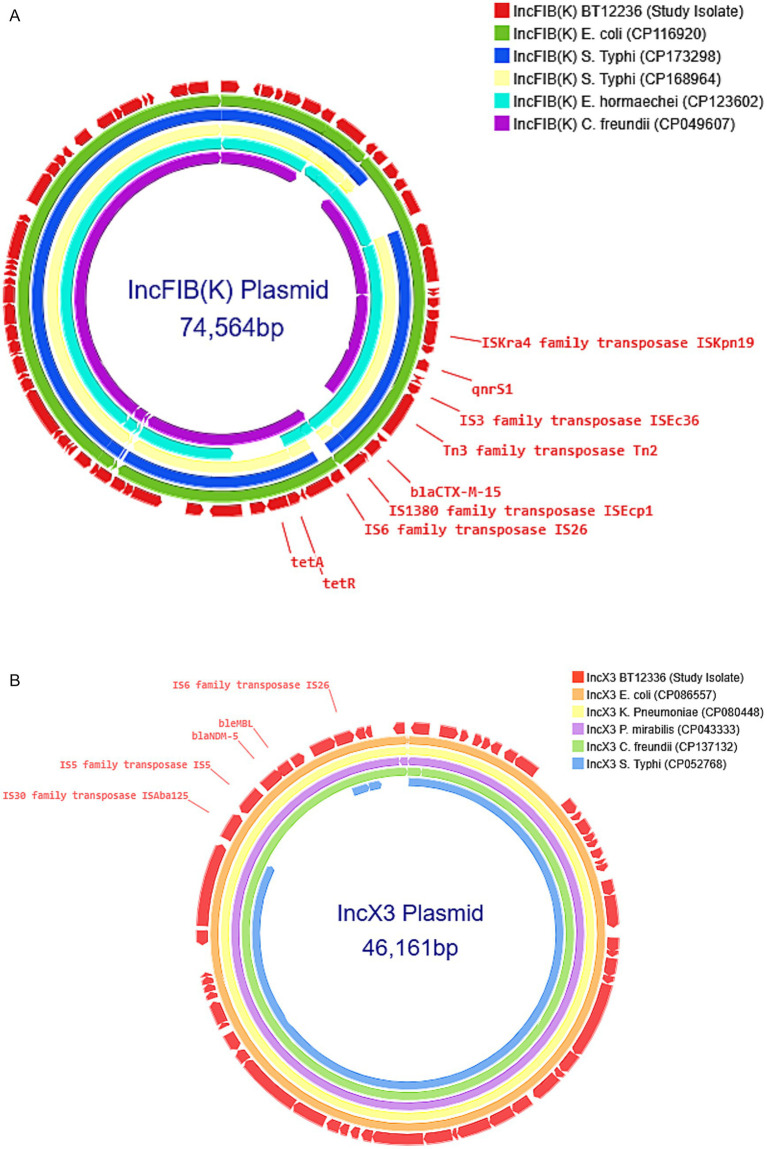
**(A)** Comparison of IncFIB(K) plasmid: The IncFIB(K) plasmid from the carbapenem-resistant *Salmonella* Typhi (CRST) isolate was BLAST-searched against the NCBI BLASTn database and compared with Enterobacteriales carrying plasmids of the same incompatibility group [(CP116920), (CP173298), (CP168964), (CP123602), (CP049607)] with 90–100% identity. The selected plasmids were then annotated and visualized using Proksee. **(B)** Comparison of IncX3 plasmid: The IncX3 plasmid from the carbapenem-resistant *Salmonella* Typhi (CRST) isolate was BLAST-searched against the NCBI BLASTn database and compared with Enterobacteriales carrying plasmids of the same incompatibility group [(CP086557), (CP080448), (CP043333), (CP137132), (CP052768)] with 90–100% identity. The selected plasmids were then annotated and visualized using Proksee.

All CRST isolates carried AMR genes located on both IncFIB(K) and IncX3 plasmids. AMR gene analysis revealed that the ~73 kb IncFIB(K) plasmid harbored *bla*_CTX-M-15_, *qnrS1*, and *tet(A)*, while the ~47 kb IncX3 plasmid carried *bla*_NDM-5_ gene. To investigate the origin of these plasmids, complete circular sequences of IncFIB(K) and IncX3 plasmids from the study isolates were BLAST-compared with previously reported plasmids in the NCBI database. The IncFIB(K) plasmid (Accession No. CP189855) showed 100% sequence identity to an *E. coli* IncFIB(K) plasmid (CP116920), as well as to plasmids previously reported in ceftriaxone-resistant *S*. Typhi isolates from Gujarat, India (CP173298 and CP168964). However, the sequence coverage was 98% for *E. coli* and 93% for *S*. Typhi, indicating that while highly similar, the plasmids are not identical. Consistent with previous reports (Thirumoorthy et al., 2025), *bla*_CTX-M-15_ gene in the IncFIB(K) plasmid was located downstream of an IS1380 family ISEcp1 element, suggesting a potential association with its mobilization (Figure 2A).

A similar BLAST analysis of the IncX3 plasmid (Accession No. CP189856) carrying *bla*_NDM-5_ revealed 100% sequence identity and query coverage with IncX3 plasmids previously reported in *E. coli* (CP086557) and *K. pneumoniae* (CP080448). Notably, the IncX3 plasmid from this study showed only 83% query coverage with the SHV-carrying IncX3 plasmid (CP052768) identified from the *S*. Typhi outbreak in Mumbai, suggesting it is genetically distinct. The *bla*_NDM-5_ was located within the characteristic genetic structure ISAba125-IS5-*bla*_NDM–5_-*ble*_MBL_-trpF-dsbC-IS26 found in IncX3 type plasmids (Figure 2B).

## Discussion

The emergence of CRST signifies a critical evolutionary escalation, building on the shift from MDR to XDR strains observed in Pakistan by 2016 (Klemm et al., 2018). Before 2016, resistance in *S*. Typhi was primarily limited to first-line antibiotics and fluoroquinolones (FQNS), with the H58 lineage dominating across South Asia and Africa (Carey et al., 2024). Within this lineage, subclades 4.3.1.1 (MDR) and 4.3.1.2 (FQNS) were distinguishable via GenoTyphi genotyping (Wong et al., 2016). Subsequent emergence of ceftriaxone-resistant and XDR *S*. Typhi in India and Pakistan, respectively, was further mapped through finer subclade resolution (Dyson and Holt, 2021; Carey et al., 2023). For example, the Sindh XDR outbreak (4.3.1.1-P1) diverged by six SNPs from its nearest contemporaries (Klemm et al., 2018), while the Vadodara outbreak in India (4.3.1.2.1) exhibited 21 SNPs from its closest relative (Thirumoorthy et al., 2025). Notably, the CRST isolates in this study have accumulated seven unique chromosomal mutations (Supplementary Table S3) while acquiring a *bla*_NDM-5_ harboring plasmid. Given the low mutation rate of *S*. Typhi (0.63 SNPs per genome per year; Wong et al., 2016), this shift appears to be a plasmid-driven phenotypic leap rather than gradual chromosomal adaptation, underscoring the role of horizontal gene transfer under selective antibiotic pressure (Rodríguez-Beltrán et al., 2021). These findings highlight unregulated antibiotic use in South Asia as a key driver of resistance evolution, emphasizing the urgent need for antimicrobial stewardship interventions.

The acquisition of diverse plasmids has played a pivotal role in the evolution of antimicrobial resistance in *S*. Typhi, driving the transition from MDR strains to emerging CRST variants. MDR *S*. Typhi strains harbored IncHI1 pST6 plasmids, almost exclusively within the H58 lineage, which became the dominant clade in South Asia and Africa (Holt et al., 2011). Resistance escalated further with the acquisition of *bla*_CTX-M-15_-carrying IncY plasmids, likely originating from *E. coli*, which facilitated the emergence of third-generation cephalosporin-resistant strains (Klemm et al., 2018). Subsequent ceftriaxone-resistant *S*. Typhi outbreaks in Mumbai and Vadodara, India, were linked to the horizontal acquisition of distinct plasmids, specifically IncX3 and IncFIB(K), respectively, likely sourced from co-circulating Enterobacteriaceae (Jacob et al., 2021; Thirumoorthy et al., 2025). This pattern indicates that *S*. Typhi has repeatedly leveraged plasmid pools from *E. coli* and *Klebsiella* spp., allowing it to bypass previously effective antibiotic therapies. The CRST isolates in this study represent the next stage in this evolutionary trajectory, having acquired an IncX3 plasmid harboring *bla*_NDM-5_ and an IncFIB(K) plasmid carrying *bla*_CTX-M-15,_ both likely derived from Enterobacteriaceae (Figure 2). Notably, the IncFIB(K) plasmid in the Vadodara outbreak differs from that found in CRST isolates, suggesting independent acquisition events rather than clonal dissemination. Similarly, the first reported carbapenem-resistant *S*. Typhi case in Peshawar, Pakistan (July 2022), carried *bla*_NDM-5_ on an IncN plasmid, again resembling plasmids found in other Enterobacteriaceae (Nizamuddin et al., 2023). The presence of multiple plasmid types (IncX3 and IncN) conferring carbapenem resistance suggests that CRST has arisen independently across different settings, rather than spreading from a single source. These findings highlight a rapidly evolving resistance landscape, where *S*. Typhi continues to integrate plasmid-borne resistance genes from Enterobacteriaceae, facilitating stepwise antibiotic resistance escalation.

In South Asia, MDR and FQNS *S*. Typhi infections are primarily treated with oral cefixime or azithromycin for outpatient cases, while intravenous ceftriaxone and azithromycin are used in combination for severe infections (Dolecek et al., 2019; Kuehn et al., 2022). For XDR or ceftriaxone resistant strains azithromycin remains the primary oral therapy for uncomplicated typhoid fever, whereas intravenous meropenem and azithromycin are recommended for severe cases (Qureshi et al., 2020; Parry et al., 2023). However, the emergence of CRST in India, co-producing *bla*_NDM_ and *bla*_CTX-M_ enzymes, renders both ceftriaxone and meropenem ineffective (Park et al., 2024). If CRST isolates remain susceptible to azithromycin (MIC ≤ 16 μg/mL), azithromycin remains the preferred oral treatment, leveraging its intracellular efficacy. Alarmingly, the first report of CRST from Peshawar, Pakistan, identified co-carriage of the *mphA* gene, conferring phenotypic resistance to azithromycin (Nizamuddin et al., 2023). In such cases, ceftazidime-avibactam + aztreonam combination therapy is recommended for severe infections (Tamma et al., 2021). Where aztreonam-avibactam is unavailable, colistin, fosfomycin, or tigecycline monotherapy may serve as alternative options (Parry et al., 2019). Given the limited clinical evidence for treating CRST, treatment decisions should be guided by individual patient factors, local resistance patterns, and expert consultation.

The rapid evolution of *S*. Typhi resistance, culminating in CRST, signals an urgent public health crisis in South Asia, where the failure of ceftriaxone and meropenem leaves severely limited treatment options for typhoid fever (Nabarro et al., 2022). Given the increasing ineffectiveness of antibiotics, preventive strategies must take priority to reduce disease burden and slow resistance evolution. One of the most effective interventions is the widespread adoption of the typhoid conjugate vaccine (TCV), which successfully curbed the Sindh XDR outbreak in Pakistan by reducing case numbers and lowering antibiotic selective pressure (Nampota-Nkomba et al., 2023; Qamar et al., 2024). Expanding TCV coverage across India and South Asia is critical to prevent CRST from becoming endemic (Mogasale et al., 2024). Beyond vaccination, strengthening water, sanitation, and hygiene (WASH) infrastructure is essential, as poor sanitation fuels *S*. Typhi’s fecal-oral transmission, sustaining high infection rates and increasing exposure to resistant strains (Luby et al., 2018). Enhanced genomic surveillance is also crucial for tracking CRST’s plasmid-driven spread and emerging resistance mutations—particularly *acrB*-R717Q/L mutations and *mphA*-mediated azithromycin resistance, as seen in Peshawar’s first CRST case (Nizamuddin et al., 2023). Finally, urgent antibiotic stewardship reforms are needed to curb the unregulated use of broad-spectrum antibiotics, a key driver of resistance escalation. Preserving azithromycin’s efficacy and ensuring restricted use of last-resort drugs like aztreonam-avibactam will be essential to maintaining effective treatment options for future cases.

In conclusion, the emergence of high-risk *S*. Typhi clones, particularly those harboring carbapenem resistance, underscores the urgent need for a comprehensive, multi-pronged strategy to contain their spread. Robust genomic surveillance is critical for tracking resistance trends, deciphering genetic evolution, and understanding transmission dynamics. Strengthening national antibiotic stewardship policies can help curb selective pressure and slow resistance escalation. Additionally, widespread typhoid conjugate vaccine (TCV) deployment can significantly reduce disease burden and limit antibiotic exposure. A coordinated global response, integrating surveillance, stewardship, and vaccination, is essential to mitigate the growing threat posed by carbapenem-resistant *S*. Typhi.

## Data Availability

The datasets presented in this study are available in online repositories. They can be retrieved under the repository name “Genomic Insights into the Emergence of Carbapenem-Resistant Salmonella Typhi harboring blaNDM-5 in Bengaluru, India” with the accession number PRJEB88734, and the plasmid sequences are available under the BioProject number PRJNA1212325.

## Footnotes

^1^ https://www.cdc.gov/mmwr/volumes/72/wr/mm7207a2.htm#:~:text=In%202019%2C%20an%20updated%20modeling%20study%20estimated,Eastern%20Mediterranean%20(187)%2C%20and%20African%20(111)%20regions

^2^ https://www.bioinformatics.babraham.ac.uk/projects/fastqc/

^3^ https://github.com/DerrickWood/kraken

^4^ https://github.com/ncbi/SKESA

^5^ https://github.com/gbouras13/hybracter

^6^ https://github.com/oschwengers/bakta

^7^ https://github.com/denglab/SeqSero2

^8^ https://cge.food.dtu.dk/services/MLST/

^9^ https://github.com/ncbi/amr

^10^ https://cge.food.dtu.dk/services/PlasmidFinder/

^11^ https://proksee.ca/

^12^ https://bridges.monash.edu/articles/dataset/Global_Typhoid_Genomics_Consortium_2022_-_Genome_Assemblies/21431883

^13^ https://github.com/tseemann/snippy

^14^ https://github.com/nickjcroucher/gubbins

^15^ https://sanger-pathogens.github.io/snp-sites/

## Author’s note

This publication is part of the Ph.D. thesis of The Tamil Nadu Dr. M.G.R. Medical University.

## Supplementary material

SUPPLEMENTARY FIGURE S1 Phylogenetic relationship of 31 Carbapenem-resistant S. Typhi isolates from India against 412 global S. Typhi strains. The maximum likelihood phylogenetic tree was constructed based on single nucleotide polymorphisms (SNPs) and mapped against the Salmonella Typhi CT18 reference strain, with an overall SNP count of 6,780. The genotypes of S. Typhi strains are represented as gradient-colored strips, while the study isolates are highlighted as red dots.

SUPPLEMENTARY FIGURE S2 Phylogenetic tree depicting the CRST subclade and its closely related isolates (*n* = 70) This figure illustrates the phylogenetic relationships among the CRST subclade and closely related Salmonella Typhi isolates sequenced between 2018 and 2020 as part of the SEFI initiative to enhance clarity and visualization of the genomic relationships.
