# Supplementary figures and images for: Emergence of carbapenem-resistant Salmonella Typhi harboring blaNDM-5 in India: genomic evidence from a multicenter study
Source: Front Microbiol. 2025 Dec 3;16:1685068. doi: 10.3389/fmicb.2025.1685068 (PMC12708597; doi:10.3389/fmicb.2025.1685068)

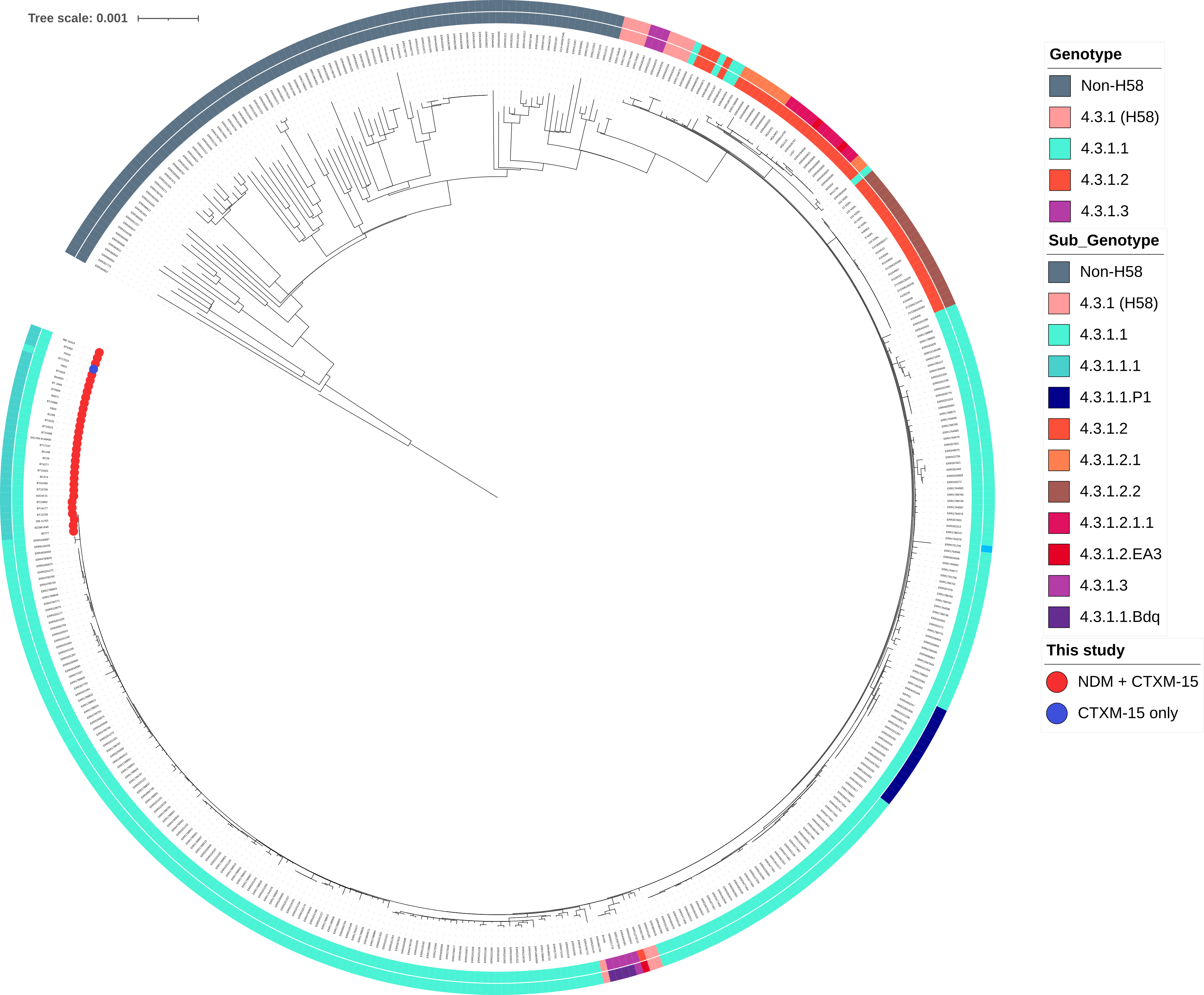

Supplement: SUPPLEMENTARY FIGURE S1 — Phylogenetic relationship of 31 Carbapenem-resistant S. Typhi isolates from India against 412 global S. Typhi strains. The maximum likelihood phylogenetic tree was constructed based on single nucleotide polymorphisms (SNPs) and mapped against the Salmonella Typhi CT18 reference strain, with an overall SNP count of 6,780. The genotypes of S. Typhi strains are represented as gradient-colored strips, while the study isolates are highlighted as red dots. [file Image_1.PNG]

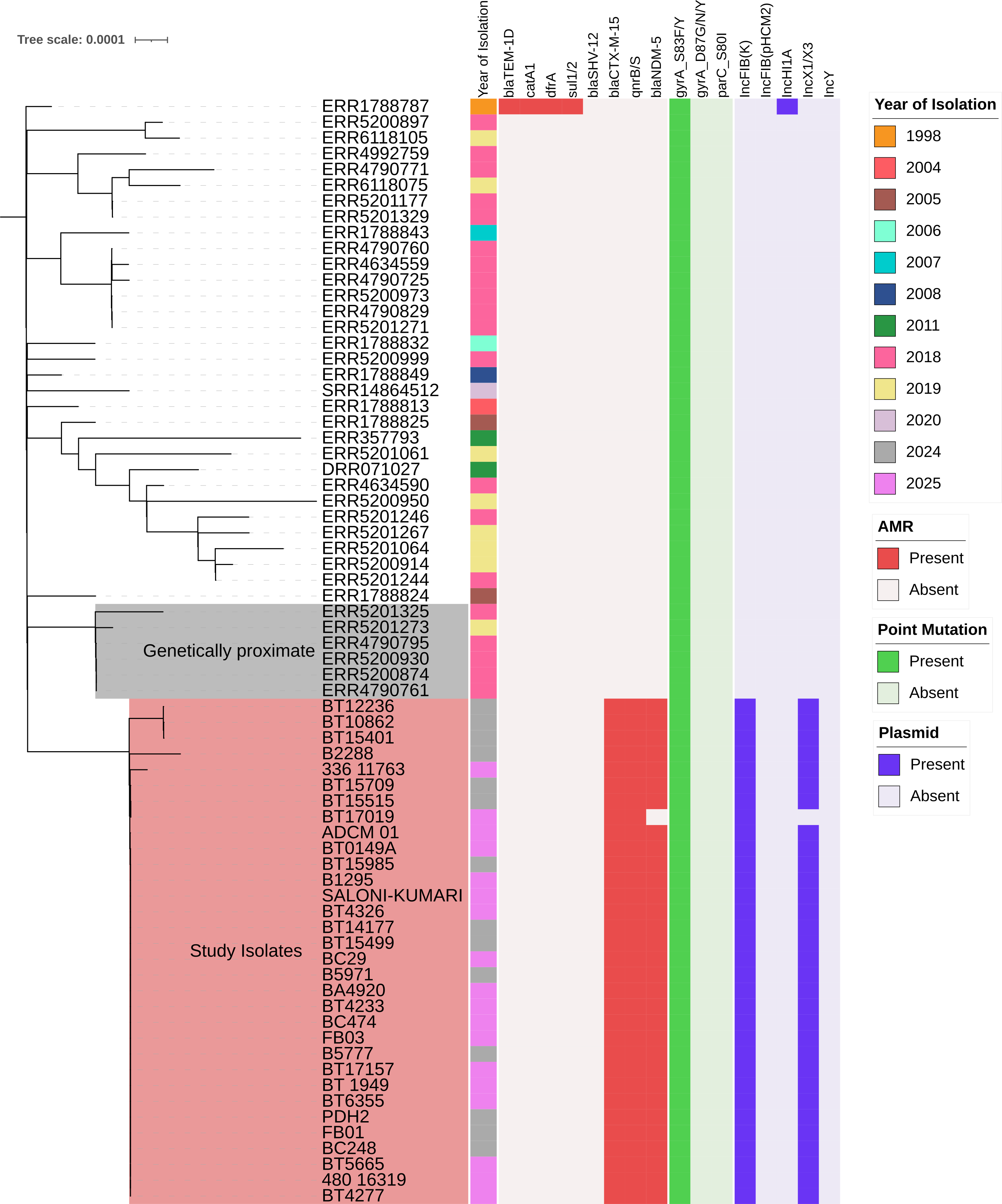

Supplement: SUPPLEMENTARY FIGURE S2 — Phylogenetic tree depicting the CRST subclade and its closely related isolates (n = 70) This figure illustrates the phylogenetic relationships among the CRST subclade and closely related Salmonella Typhi isolates sequenced between 2018 and 2020 as part of the SEFI initiative to enhance clarity and visualization of the genomic relationships. [file Image_2.PNG]
